# Supplementary material for: Support for, and perceived effectiveness of, the UK soft drinks industry levy among UK adults: cross-sectional analysis of the International Food Policy Study
Source: BMJ Open. 2019 Mar 3;9(3):e026698. doi: 10.1136/bmjopen-2018-026698 (PMC6429875; doi:10.1136/bmjopen-2018-026698)
Supplement: Author's manuscript [file bmjopen-2018-026698.draft_revisions.pdf]

BMJ Open is committed to open peer review. As part of this commitment we make the peer review history of every article we publish publicly available.

When an article is published we post the peer reviewers' comments and the authors' responses online. We also post the versions of the paper that were used during peer review. These are the versions that the peer review comments apply to.

The versions of the paper that follow are the versions that were submitted during the peer review process. They are not the versions of record or the final published versions. They should not be cited or distributed as the published version of this manuscript.

BMJ Open is an open access journal and the full, final, typeset and author-corrected version of record of the manuscript is available on our site with no access controls, subscription charges or pay-per-view fees (http://bmjopen.bmj.com).

If you have any questions on BMJ Open's open peer review process please email info.bmjopen@bmj.com

BMJ Open

SUPPORT FOR, AND PERCEIVED EFFECTIVENESS OF, THE UK SOFT DRINKS INDUSTRY LEVY AMONGST UK ADULTS: CROSS-SECTIONAL ANALYSIS OF THE INTERNATIONAL FOOD POLICY SURVEY

Journal:	*BMJ Open*
Manuscript ID	bmjopen-2018-026698
Article Type:	Research
Date Submitted by the Author:	14-Sep-2018
Complete List of Authors:	Pell, David; University of Cambridge, Centre for Diet & Activity Research, MRC Epidemiology Unit<br>Penney, Tarra; University of Cambridge, Centre for Diet & Activity Research, MRC Epidemiology Unit<br>Hammond, D; University of Waterloo,<br>Vanderlee, Lana; University of Waterloo<br>White, Martin; University of Cambridge, Centre for Diet & Activity Research, MRC Epidemiology Unit<br>Adams, J; University of Cambridge, Centre for Diet & Activity Research
Keywords:	taxation, soda tax, PUBLIC HEALTH, attitudes, NUTRITION & DIETETICS

SCHOLARONE™
Manuscripts

For peer review only

SUPPORT FOR, AND PERCEIVED EFFECTIVENESS OF, THE UK SOFT DRINKS INDUSTRY LEVY AMONGST UK ADULTS: CROSS-SECTIONAL ANALYSIS OF THE INTERNATIONAL FOOD POLICY SURVEY

David Pell,[1] *Tarra Penney,*[1] *David Hammond,*[2] *Lana Vanderlee,*[2] *Martin White,*[1] *Jean Adams*[1*]

[1]Centre for Diet & Activity Research, MRC Epidemiology Unit, University of Cambridge, Cambridge, UK

[2]School of Public Health & Health Systems, University of Waterloo, Waterloo, Ontario, Canada

***Corresponding author:** Jean Adams, Centre for Diet & Activity Research, MRC Epidemiology Unit, University of Cambridge School of Clinical Medicine, Box 285 Institute of Metabolic Science, Cambridge Biomedical Campus, Cambridge, UK, CB2 0QQ. Email: jma79@medschl.cam.ac.uk Tel: +44 (0) 1223 769 142

Main text word count: 3039

For peer review only

ABSTRACT

Objectives To answer four questions: What are attitudes, knowledge and social norms around sugar-sweetened beverages (SSBs)? What are current levels of trust in messages on SSBs? What is current support for, and perceived effectiveness of, the UK Soft Drinks Industry Levy (SDIL)? What is the association between attitudes, knowledge, social norms, trust, SSB consumption and socio-demographic factors; and support for, and perceived effectiveness of, the SDIL?

Design Cross-sectional on-line survey.

Setting UK

Participants UK respondents to the 2017 International Food Policy Study aged 18-64 years who provided information on all variables of interest (n=3104).

Outcome measures Self-reported perceived effectiveness of, and support for, the SDIL.

Results Most participants supported the SDIL (70%), believed it will be effective (71%), had a positive attitude to SSBs (62%), had knowledge of the link between SSBs and obesity (90%), and trusted messages from health experts (61%), but not those from the food and beverage industry (73%). Nearly half (46%) had negative social norms about drinking SSBs. In adjusted models, older age, non-consumption of SSBs, social norms to not drinks SSBs, knowledge of the link between SSBs and obesity and trust in health expert messages were associated with greater support for the SDIL, whereas having dependent children and trusting messages from the food and beverage industry were associated with less support. In adjusted models, older age was associated with lower perceived effectiveness of the SDIL, whereas social norms to not drink SSBs, negative attitudes to SSBs, and trusting messages from health experts and the food and beverage industry were associated with greater perceived effectiveness.

Conclusions There was strong support for the SDIL and belief that it will be effective. Those with more 'public health' orientated norms and trust were generally more likely to support the SDIL or believe that it will be effective.

Keywords: taxation, soda tax, public health, attitudes, nutrition & dietetics

For peer review only

STRENGTHS AND LIMITATIONS OF THE STUDY

- We used a large, population representative sample.
- We were careful to present the SDIL as an intervention targeted at manufacturers rather than consumers, with revenues ear-marked for health-promotion activities.
- This is a cross-sectional analysis and we cannot be sure of the direction of causation between putative explanatory variables and outcomes.
- Whilst all have strong face validity, we have not explored other aspects of validity or reliability of any of the measures used; in many cases it would be hard to know what the 'gold standard' measure should be.
- A high proportion of participants who completed the survey were included in the analysis, but we do not know the response rate.

For peer review only - http://bmjopen.bmj.com/site/about/guidelines.xhtml

INTRODUCTION

In his March 2016 Budget Statement, the UK Chancellor of the Exchequer (minister of finance) announced a soft drinks industry levy (SDIL) to be implemented in April 2018.[1] The levy is imposed on industries importing or manufacturing sugar-sweetened beverages (SSBs) and includes two 'tiers'. Drinks with ≥8g of sugar per 100ml are charged £0.24 per litre and those with ≥5g but <8g per 100ml are charged £0.18 per litre. Alcoholic drinks, milk-based drinks and pure fruit juices are exempt irrespective of sugar content. The Chancellor stated that revenue raised would be spent on school sport and school breakfast clubs. An explicit aim of announcing the levy two years in advance of implementation, and defining two levy tiers, was to provide time for manufacturers to reformulate.[1] The nature and intent of the SDIL makes it unique amongst international SSB taxes.

The success or failure of policy interventions is often the result of actions and reactions by many stakeholders including government, civil society, industry, the health sector and consumers. In particular support for the SDIL may both be influenced by the SDIL and modify its effectiveness.

More intrusive public health interventions, like food taxes, generally receive lower levels of public support than less intrusive ones, like information giving.[2] Support for hypothetical SSB taxes has been reported to range from 36-60%.[3-17] Support generally increases when it is proposed that the revenue raised would be used for health promoting purposes.[11 12 18 19]

Previous work has explored differences in support for SSB taxes according to participant socio-demographic characteristics, but findings are not consistent. For example, support has been varyingly reported as higher in younger people,[3 17 20] higher in older people,[18] and not associated with age.[5 10] Associations between support for SSB taxes and both SSB consumption and markers of socio-economic position are similarly variable.[3 5 8 16 18 20] Fewer studies have explored psychological correlates of support for SSB taxes, such as attitudes, social norms, knowledge and trust. Those who felt that SSBs were a major (but not minor) contributor to childhood obesity in the USA were more likely to support an SSB tax.[5] Although trust in government was not associated with support in either the UK or USA,[10] more favourable assessments of soft drinks companies were associated with lower support in the USA.[20]

One reason for low support for SSB taxes commonly found in qualitative work is low perceived effectiveness of small changes in price.[6 7 11 12 19] Perceived effectiveness is less studied in quantitative studies, but has been found to range from 39-58%.[5 12 18] Perceived effectiveness was

found to be an important correlate of support in one quantitative study,[10] and has also been reported to be higher in older people and those with more education; but lower in those consuming more SSBs.[18]

The great majority of work in this area has focused on hypothetical taxes. As support for more intrusive public health interventions often increases after implementation,[2] support for hypothetical SSB taxes may misrepresent support for 'real' taxes. To date, we are aware of only one study that has explored public perceptions of a real tax.[18] This study was conducted in France where an excise tax applies to all sweetened drinks, including those sweetened with artificial sweeteners. Given the difference between the French tax and SSB taxes, which are more specific to drinks sweetened with sugar, the French findings may not be generalisable.

In this study we explored both socio-demographic and psychological correlates of support for, and perceived effectiveness of, a real SSB tax. Using data from UK adults collected 20 months after announcement and four months before implementation of the SDIL, our specific research questions were: 1. What are current attitudes, knowledge and social norms around SSBs? 2. What are current levels of trust in messages on SSBs from different institutions? 3. What is current support for, and perceived effectiveness of, the SDIL? 4. What is the association between attitudes, knowledge, social norms, trust, SSB consumption and socio-demographic factors; and support for, and perceived effectiveness of, the SDIL?

METHODS

Sampling, recruitment and data collection

Data were from UK participants in Wave 1 of the International Food Policy Study, conducted in Australia, Canada, Mexico, the United Kingdom and the United States. Data were collected via self-completed web-based surveys in December 2017 with adults aged 18-64 years. Respondents were recruited through Nielsen Consumer Insights Global Panel and their partners' panels. Email invitations (with a unique link) were sent to a random sample of panelists (after targeting for age and country criteria); panelists known to be ineligible were not invited. The mean survey time across countries was 33 minutes.

Respondents provided consent prior to completing the survey. Respondents received remuneration in accordance with their panel's usual incentive structure (e.g., points-based or monetary rewards, or chances to win prizes). The study was reviewed by and received ethics clearance through a University of

For peer review only - http://bmjopen.bmj.com/site/about/guidelines.xhtml

Waterloo Research Ethics Committee (ORE# 21460). A full description of the study methods can be found in the International Food Policy Study: Technical Report – Wave 1 (2017) at www.foodpolicystudy.com/methods.

Variables used in the analysis

The variables used in the analysis, the survey items they were derived from, response options and how response options were collapsed for analysis are described in Table 1.

Alongside single-item measures of attitudes, knowledge, and social norms related to sugary drinks; we included single items measures of trust in advice on sugary drinks from health experts and the food and beverage industry; and single item measures of support for, and perceived effectiveness of, the SDIL. As previous research has indicated that the acceptability of food taxes varies with the stated intentions of these,[11 12 18 19] we included a preamble to the questions about support for, and perceived effectiveness of, the SDIL outlining the intention of the levy and the stated use of revenue generated.

Sociodemographic variables considered were age in years, sex at birth, whether or not participants had children and socio-economic position. Parental status was a potentially important variable because the SDIL is included as a flagship component of England's Childhood Obesity Plan and has particularly been framed in terms of potential benefits to children.[1 21] Socio-economic position was measured using participants' highest educational qualification and perceived income sufficiency.

The BFQ is a 7-day food record that assesses consumption for 17 beverage categories, including caloric and non-caloric beverages.[22] For each beverage category, respondents report the number of drinks and the usual portion size, using category-specific images of beverage containers, adapted from the ASA24 dietary recall.[23] Participants who reported any consumption of regular fizzy drinks (including alcoholic drinks that contained regular fizzy drinks as a mixer), sweetened fruit drinks, sports drinks, or energy drinks over the previous seven days were considered SSB consumers in the analysis.

Inclusion criteria

UK resident participants in wave 1 of the International Food Policy Survey, aged 18-64 years, who correctly responding to a data integrity question in which participants were asked to identify the current month, and provided usable information on all other variables of interest were included in the analysis.

For peer review only - http://bmjopen.bmj.com/site/about/guidelines.xhtml

Table 1. Description of items and response options used in the analysis

		Response options	
**Concept**	**Item wording (where applicable)**	**All**	**Used in analysis**
Age	How old are you?	In years	In years
Sex	What sex were you assigned at birth, meaning on your original birth certificate?	Female	Female
		Male	Male
Education	What is the highest level of education you have completed?	Qualifications not listed below, free-text equivalents, Don't Know, Refuse to answer	≤School leaving
		NVQ Level 4-5, HNC, HND, RSA Higher Diploma, BTEC Higher Level, Degree, Higher Degree, free-text equivalents	>School leaving
Income sufficiency	How easy is it to make ends meet?	Neither easy nor difficult, Difficult, Very difficult, Don't know, Refuse to answer	Not easy
		Very easy, Easy	Easy
Children	Do you have any children (including step-children or adopted children) under the age of 18?	No, Don't know, Refuse to answer	No
		Yes	Yes
SSB consumption	[Calculated from Beverage Frequency Questionnaire: reported consumption over last 7 days]	Any consumption of non-diet Fizzy drinks, Sweetened fruit juice drinks, Regular sports drinks, Regular energy drinks, or Spirits with mixers that have calories	Consumers
		No consumption of above	Non-consumers
Social norms	People important to me try not to drink sugary drinks	Neither agree nor disagree, Disagree, Strongly disagree, Don't know, Refuse to answer	Not agree
		Strongly agree, Agree	Agree
Attitudes	Sugary drinks taste good	Strongly agree, Agree	Agree
		Neither agree nor disagree, Disagree, Strongly disagree, Don't know, Refuse to answer	Not agree
Knowledge	Frequently drinking sugary drinks increases the risk of obesity	False, Don't know, Refuse to answer	Not true
		True	True
Expert trust	I trust messages from health experts on sugary drinks	Neither agree nor disagree, Disagree, Strongly disagree, Don't know, Refuse to answer	Not agree
		Strongly agree, Agree	Agree
Industry trust	I trust messages from the food and beverage industry on sugary drinks?	Neither agree nor disagree, Disagree, Strongly disagree, Don't know, Refuse to answer	Not agree
		Strongly agree, Agree	Agree
Support	In 2018 a new sugary drink tax will be introduced in the UK. This aims to encourage manufacturers to reduce the sugar in drinks. The money will be spent on breakfast clubs, and sports in primary schools. Do you support or oppose this policy?	Strongly support, Support	Support
		Oppose, Strongly oppose, Don't know, Refuse to answer	Oppose
Effectiveness	Preamble as above. How effective do you think these kinds of policies are?	Somewhat effective, Mostly effective, Very effective	Effective
		Not at all effective, Don't know, Refuse to answer	Not effective

For peer review only

For peer review only - http://bmjopen.bmj.com/site/about/guidelines.xhtml

Analysis

Data were weighted with post-stratification sample weights constructed using population estimates from the UK census based on age group, sex and region. These sample weights were used throughout the analysis to reduce the effects of non-response and selection bias and return the sample to population representativeness.

Descriptive statistics were used to quantify all variables of interest. Logistic regression models were fitted to explore associations between other variables and support for, and perceived effectiveness of, the SDIL. We used separate models to explore support for the SDIL and perceived effectiveness of the SDIL where support for, or perceived effectiveness of, the SDIL were the outcome variables and all other variables were included as explanatory variables. Unless otherwise noted, adjusted odds ratios (and 95% confidence intervals) of support for, or perceived effectiveness of, the SDIL are presented adjusted for all other variables included.

RESULTS

Of 4276 who took part in the in the UK arm of the International Food Policy Survey in December 2017, 4047 (95%) correctly responded to the data integrity question. Of these, 3104 (77%) provided complete data on all variables of interest and were included the analysis.

Characteristics of the analytical sample (after applying survey weights) are described in Table 2. Participants had a mean age of 38 (standard deviation 13) years, with a good balance across sex at birth (48% female). The highest level of education that most participants had achieved was the equivalent of school-leaving or lower and around two thirds (61%) did not find it easy to make ends meet. Just over one third (37%) of participants had children under the age of 18 years, and just less than half (47%) reported consuming SSBs in the last seven days.

Around half of participants (54%) agreed that people important to them try not to drink SSBs (social norms), around two thirds (62%) that SSBs taste good (attitudes), and 90% believed that frequently consuming SSBs increases the risk of obesity (knowledge). Whilst more than half (61%) of respondents trusted messages from health experts on SSBs, only one quarter (27%) trusted messages from the food and beverage industry.

For peer review only - http://bmjopen.bmj.com/site/about/guidelines.xhtml

Table 3 shows the results of logistic regression analyses of associations between socio-demographics, social norms, attitudes, knowledge and trust, and perceived support for, and effectiveness of, the SDIL – adjusted for all other variables in the models.

In adjusted models, older participants were more likely to support the SDIL, but were less likely to consider it effective. Those with dependent children and those who trusted messages from the food and beverage industry on sugary drinks were less likely to support the SDIL. Non-consumers of SSBs, those with social norms to not drinks SSBs, those with knowledge of the association between SSBs and obesity, and those who trust messages from health experts on sugary drinks were more likely to support the SDIL than other. Those with high social norms around not drinking SSBs, less positive attitudes to sugary drinks, and those who trusted messages on sugary drinks from health experts and from the food and beverage industry were more likely to consider the SDIL would be effective. There were no differences in support for or perceived effectiveness of the SDIL by sex, education or perceived income sufficiency.

For peer review only - http://bmjopen.bmj.com/site/about/guidelines.xhtml

Table 2. Weighted characteristics of UK participants in the International Food Policy Survey, Dec 2017

Concept	Question wording (where applicable)	Response category	n	%
Sex	What sex were you assigned at birth, meaning on your original birth certificate?	Female	1497	48
		Male	1607	52
Education	What is the highest level of education you have completed?	A-Levels or lower	1896	61
		> A-Levels	1208	39
Income sufficiency	How easy is it to make ends meet?	Not easy	1905	61
		Easy	1199	39
Children	Do you have any children (including step-children or adopted children) under the age of 18?	No	1963	63
		Yes	1141	37
SSB consumption	Consumed regular fizzy drinks, sweetened fruit drinks, sports drinks, energy drinks in last 7 days	Consumers	1473	47
		Non-consumers	1631	53
Social norms	People important to me try not to drink sugary drinks	Not agree	1416	46
		Agree	1688	54
Attitudes	Sugary drinks taste good	Agree	1938	62
		Not agree	1166	38
Knowledge	Frequently drinking sugary drinks increases the risk of obesity	Not true	322	10
		True	2782	90
Expert trust	I trust messages from health experts on sugary drinks	Not agree	1213	39
		Agree	1891	61
Industry trust	I trust messages from the food and beverage industry on sugary drinks	Not agree	2267	73
		Agree	837	27
Support	In 2018 a new sugary drink tax will be introduced in the UK. This aims to encourage manufacturers to reduce the sugar in drinks. The money will be spent on breakfast clubs, and sports in primary schools. Do you support or oppose this policy?	Support	2167	70
		Oppose	937	30
Effectiveness	Preamble as above. How effective do you think these kinds of policies are?	Effective	2214	71
		Not effective	890	29

For peer review only

For peer review only - http://bmjopen.bmj.com/site/about/guidelines.xhtml

Table 3. Adjusted* odds ratios (95% confidence intervals) of characteristics associated with support for, and perceived effectiveness of, the SDIL

Concept	Question wording (where applicable)	Response category	Support SDIL, n (%)	Adjusted OR (95% CI) of SDIL support	SDIL effective, n (%)	Adjusted OR (95% CI) of SDIL effectiveness
Age	How old are you?	Years	NA	**1.01 (1.00 to 1.02)**	NA	**0.99 (0.98 to 0.99)**
Sex	What sex were you assigned at birth, meaning on your original birth certificate?	Female	1083 (72)	Reference	1074 (72)	Reference
		Male	1084 (67)	(0.72 to 1.05)	1140 (71)	1.03 (0.85 to 1.25)
Education	What is the highest level of education you have completed?	A-Levels or lower	1297 (68)	Reference	1352 (71)	Reference
		> A-Levels	870 (72)	1.03 (0.85 to 1.26)	862 (71)	0.90 (0.73 to 1.10)
Income sufficiency	How easy is it to make ends meet?	Not easy	1300 (68)	Reference	1351 (71)	Reference
		Easy	867 (72)	1.01 (0.83 to 1.24)	862 (72)	1.02 (0.83 to 1.25)
Dependent children	Do you have any children (including step-children or adopted children) under the age of 18?	No	1425 (73)	Reference	1369 (70)	Reference
		Yes	741 (65)	**0.81 (0.67 to 0.99)**	845 (74)	1.16 (0.94 to 1.43)
SSB consumption	Consumed regular fizzy drinks, sweetened fruit drinks, sports drinks, energy drinks in last 7 days	Consumers	925 (63)	Reference	1030 (70)	Reference
		Non-consumers	1241 (76)	**1.57 (1.28 to 1.91)**	1184 (73)	1.21 (0.99 to 1.48)
Social norms	People important to me try not to drink sugary drinks	Not agree	901 (64)	Reference	952 (67)	Reference
		Agree	1265 (75)	**1.39 (1.15 to 1.70)**	1262 (75)	**1.25 (1.03 to 1.53)**
Attitudes	Sugary drinks taste good	Agree	1304 (67)	Reference	1355 (70)	Reference
		Not agree	863 (74)	1.10 (0.89 to 1.36)	859 (74)	**1.31 (1.07 to 1.61)**
Knowledge	Frequently drinking sugary drinks increases the risk of obesity	Not true	142 (44)	Reference	217 (67)	Reference
		True	2025 (73)	**2.34 (1.74 to 3.16)**	1997 (72)	1.06 (0.77 to 1.45)
Expert trust	I trust messages from health experts on sugary drinks	Not agree	748 (62)	Reference	753 (62)	Reference
		Agree	1419 (75)	**2.01 (1.63 to 2.49)**	1461 (77)	**1.86 (1.51 to 2.28)**
Industry trust	I trust messages from the food and beverage industry on sugary drinks	Not agree	1636 (72)	Reference	1547 (68)	Reference
		Agree	531 (63)	**0.55 (0.44 to 0.69)**	667 (80)	**1.37 (1.08 to 1.75)**

*All results are adjusted for all other variables listed; SDIL = soft drinks industry levy; **BOLD** indicates statistically significant at the p<0.05 level

For peer review only - http://bmjopen.bmj.com/site/about/guidelines.xhtml

DISCUSSION

Summary of findings

To our knowledge, this is the first study of a range of socio-demographic, consumption and psychological correlates of both support for, and perceived effectiveness of, an SSB tax. Unlike previous studies, our research was conducted in the context of a 'real', rather than hypothetical, SSB tax. We found that the majority of UK adults aged 18-64 years were supportive of the SDIL and believe it will be effective, have a positive attitude to SSBs, have good knowledge about the links between SSBs and obesity, and trust messages from health experts, but not the food and beverage industry, about sugary drinks. Around half reported social norms about not drinking SSBs.

Social norms towards not consuming SSBs and trusting health expert messages on SSBs were both associated with greater support for and perceived effectiveness of the SDIL. In addition, having dependent children and trusting messages from the food and beverage industry on sugary drinks were associated with less support for the SDIL, whilst older age, not consuming SSBs and knowledge of the link between sugary drinks and obesity were associated with greater support. Older age was associated with lower perceived effectiveness of the SDIL, and more negative attitudes towards sugary drinks were associated with greater perceived effectiveness. There were no associations between gender, education or income sufficiency and either support for, of perceived effectiveness of, the SDIL.

Strengths and weaknesses of methods

Key strengths of the analysis are the use of a large, population representative, sample; inclusion of a range of socio-demographic, consumption and psychological variables; and the context of a 'real' SSB tax announced 20 months before data collection (although not implemented until four months after). Given previous findings that support is greater when revenues are used for health-promoting activities,[11 12 18 19] we were careful to present the SDIL as an intervention targeting manufacturers rather than consumers, with revenues ear-marked for health-promotion activities. Social desirability bias may be less likely to occur in more anonymous settings such as on-line surveys.[24]

Participants were not recruited using probability-based sampling meaning the findings do not provide nationally representative estimate, although this was reduced by applying sampling weights. The results are, therefore, likely to be generalizable to the UK, but may not be more widely generalizable. This is a cross-sectional analysis and we cannot be sure of the direction of causation between putative explanatory variables and outcomes. Nor have we explored more complicated causal networks linking

For peer review only - http://bmjopen.bmj.com/site/about/guidelines.xhtml

the variables included. All variables were self-reported. Whilst all have strong face validity, we have not explored other aspects of validity or reliability of any of the measures used. However, all were derived from existing instruments in some cases it would be hard to know what a 'gold standard' measure should be. Although a high proportion of participants who completed the survey were included in the analysis, we do not know what proportion of those invited to participate were included.

Comparison to previous results and interpretation of findings

Most people in our survey (90%) knew that there was an association between SSB consumption and obesity. This reflects previous findings where 89-91% agreed that SSB consumption increased the risk of obesity.[3 5] Despite this, there were also high positive attitudes towards SSBs with almost two-third of respondents agreeing that sugary drinks taste good, and less than half had social norms about not drinking SSBs. In the UK, SSBs appear to remain a pleasurable and positive part of life, despite their known health harms.

Similar to previous research which found that only 30% of Americans gave favourable ratings to soda companies,[20] we found low levels of trust in messages about SSBs from the food and beverage industry. Levels of trust in similar messages from health experts were higher, but still less than two thirds. Low levels of trust in experts may reflect a general public mistrust of nutritional epidemiology.[25]

Despite less than perfect trust in messages about SSBs from health experts, there was a high level of support for the SDIL (70%) and even higher belief that it would be effective (71%). This is higher than previous research which, as far as we are aware, reports maximum support of 60%.[8 26] Even in the context of an existing tax on sweetened drinks in France, only 49% supported the tax.[18] The high level of support we found may reflect the combined effect of previous findings that support for public health interventions often increases after implementation,[2] and that support for SSB taxes is often greater when revenues are used for health-promoting activities.[11 12 18 19 26 27] Although the SDIL had not been implemented at the time of data collection, impending implementation had been known of for 20 months. Further, we were careful to inform participants that SDIL revenues would be spent on school breakfast clubs and sports activities. In addition, the SDIL is unique in being targeted at manufacturers rather than consumers, and intended to promote reformulation rather than necessarily reduce consumption.[1] Previous qualitative work has found that those who do not support generic SSB taxes often cite excessive personal taxation and government intrusion into individual's lives as reasons for this.[11 28] This is much less applicable to the SDIL than to consumer-facing SSB taxes.

For peer review only - http://bmjopen.bmj.com/site/about/guidelines.xhtml

Low acceptability of SSB taxes has previously been ascribed to a perception that they are unlikely to achieve significant behaviour change or public health benefit.[11 28] Previous research has reported perceived effectiveness (to improve population health or decrease SSB consumption) in the range of 39-58%.[5 12 18] In contrast, we found much higher levels of perceived effectiveness (71%). This may again reflect the unique nature of the SDIL with an explicit intention to change manufacturer, rather than consumer, behaviour – and our focus on effects on industry, rather than consumer, behaviour.

The pattern of associations between attitudes, social norms, trust and support for, and perceived effectiveness of, the SDIL are, for the most part, intuitive. It might be expected that non-consumers, who are less likely to be negatively financially effected by the tax, would be more supportive. In other contexts, those who stand to gain most from financial incentive interventions are most supportive.[29] Social norms to not drink sugary drinks, negative attitudes towards sugary drinks, greater knowledge about the health harms of sugary drinks, greater trust in health experts and less trust in the food and beverage industry all reflect more 'public health' orientated patterns that would be expected to be associated with greater support for, or perceived effectiveness, of the SDIL. As described above, previous research on the association between psychological variables and support for, and perceived effectiveness of, SSB taxes is sparse.

We did not find that gender or markers of socio-economic position were associated with support for, or perceived effectiveness of, the SDIL in mutually adjusted models. This reflects some, but not all, previous findings.[5 8 10 18 20] Unlike most previous work we included a wide range of socio-demographic, consumption and psychological variables in mutually adjusted models and it may be that gender or socio-economic differences operate entirely through the other variables included in our models.

Implications of findings

Many structural public health policies require government action, which may be limited by perceptions concerning public acceptability of such policies – often uninformed by evidence. Greater understanding of public acceptability of a range of structural public health policies, and how this changes over time and the course of implementation, may help to develop strategies to address public concerns and build public support.

For peer review only - http://bmjopen.bmj.com/site/about/guidelines.xhtml

CONCLUSIONS

UK adults tend to have positive attitudes to SSBs and do not necessarily have strong social norms about not drinking SSBs, but they generally recognise the link between SSB consumption and obesity. Trust in messages about SSBs from the food and drinks industry is low, but trust in these messages from health experts is not universally high. There was strong support for the SDIL and belief that it will be effective. Those with more 'public health' orientated norms and trust were generally more likely to support the SDIL or believe that it will be effective.

For peer review only

FUNDING

Funding for the International Food Policy Study was provided by the Canadian Institutes of Health Research (CIHR; operating grant). Additional support was provided by a CIHR – Public Health agency of Canada (PHAC) Applied Public Health Research Chair. The study has no affiliations with the food industry. The analyses reported in this paper were supported by The Health Foundation. JA & MW are supported by the Centre for Diet and Activity Research (CEDAR), a UKCRC Public Health Research Centre of Excellence. Funding from the British Heart Foundation, Cancer Research UK, Economic and Social Research Council, Medical Research Council, the National Institute for Health Research, and the Wellcome Trust, under the auspices of the UK Clinical Research Collaboration, is gratefully acknowledged (grant number MR/K023187/1). Views expressed in this paper are those of the authors and not necessarily those of the above named funders.

COMPETING INTERESTS

None declared

AUTHOR STATEMENT

JA, TP & MW conceived the idea for this paper. DP analysed the data. JA drafted the manuscript. All authors read and provided critical comments on the manuscript and approved the final version. DH conceived the idea for the IFPS, secured funding and developed the first draft of survey. TP led the further development of the UK survey instrument, with input from JA and MW.

DATA SHARING

Data is available directly from the International Food Policy Study team on reasonable request (see www.foodpolicystudy.com).

For peer review only - http://bmjopen.bmj.com/site/about/guidelines.xhtml

REFERENCES

1. Gauke D. Budget 2016. London: HM Treasury, 2016.

2. Diepeveen S, Ling T, Suhrcke M, Roland M, Marteau T. Public acceptability of government intervention to change health-related behaviours: a systematic review and narrative synthesis. BMC Public Health 2013;**13**:756

3. Rivard C, Smith D, McCann SE, Hyland A. Taxing sugar-sweetened beverages: a survey of knowledge, attitudes and behaviours. Public Health Nutr. 2012;**15**(8):1355-61 doi: 10.1017/s1368980011002898[published Online First: Epub Date]|.

4. Barry CL, Niederdeppe J, Gollust SE. Taxes on sugar-sweetened beverages: results from a 2011 national public opinion survey. Am. J. Prev. Med. 2013;**44**(2):158-63 doi: 10.1016/j.amepre.2012.09.065[published Online First: Epub Date]|.

5. Donaldson EA, Cohen JE, Rutkow L, Villanti AC, Kanarek NF, Barry CL. Public support for a sugar-sweetened beverage tax and pro-tax messages in a Mid-Atlantic US state. Public Health Nutr. 2015;**18**(12):2263-73

6. Jou J, Niederdeppe J, Barry CL, Gollust SE. Strategic messaging to promote taxation of sugar-sweetened beverages: lessons from recent political campaigns. Am. J. Public Health 2014;**104**(5):847-53 doi: 10.2105/ajph.2013.301679[published Online First: Epub Date]|.

7. Niederdeppe J, Gollust SE, Jarlenski MP, Nathanson AM, Barry CL. News coverage of sugar-sweetened beverage taxes: pro- and antitax arguments in public discourse. Am. J. Public Health 2013;**103**(6):e92-8 doi: 10.2105/ajph.2012.301023[published Online First: Epub Date]|.

8. Simon PA, Chiang C, Lightstone AS, Shih M. Public Opinion on Nutrition-Related Policies to Combat Child Obesity, Los Angeles County, 2011. Prev. Chronic. Dis. 2014;**11**:E96 doi: 10.5888/pcd11.140005[published Online First: Epub Date]|.

9. Somerville C, Marteau TM, Kinmonth AL, Cohn S. Public attitudes towards pricing policies to change health-related behaviours: a UK focus group study. The European Journal of Public Health 2015 doi: 10.1093/eurpub/ckv077[published Online First: Epub Date]|.

10. Petrescu D, Hollands G, Couturier D, Ng Y, Marteau T. Public acceptability in the UK and USA of nudging to reduce obesity: The example of reducing sugar-sweetened beverages consumption. PLoS ONE 2016;**11**(6):1-18

11. Thomas-Meyer M, Mytton O, Adams J. Public responses to proposals for a tax on sugar-sweetened beverages: A thematic analysis of online reader comments posted on major UK news websites. PLoS ONE 2017;**12**(11):e0186750 doi: 10.1371/journal.pone.0186750[published Online First: Epub Date]|.

12. Timpson H, Lavin R, Hughes L. Exploring the Acceptability of a Tax on Sugar-Sweetened Beverages: Insight Work. Liverpool: Centre for Public Health, Liverpool John Moores University, 2013.

13. Beeken RJ, Wardle J. Public beliefs about the causes of obesity and attitudes towards policy initiatives in Great Britain. Public Health Nutr. 2013;**16**(12):2132-7 doi: 10.1017/s1368980013001821[published Online First: Epub Date]|.

For peer review only

For peer review only - http://bmjopen.bmj.com/site/about/guidelines.xhtml

14. Cancer Research UK, UK Health Forum. Short and sweet: why the government should introduce a sugary drinks tax in the UK. London, 2016.

15. ComRes. Daily Mail sugar tax poll. Secondary Daily Mail sugar tax poll 2015. http://www.comres.co.uk/polls/daily-mail-sugar-tax-poll/.

16. Ipsos Mori Social Research Institute. Great Britain: The way we live now. Understanding Society 2013;**December 2013**

17. Curry LE, Rogers T, Williams P, Homsi G, Willett J, Schmitt CL. Public Attitudes and Support for a Sugar-Sweetened Beverage Tax in America's Heartland. Health Promot. Pract. 2017:1524839917709759 doi: 10.1177/1524839917709759[published Online First: Epub Date]|.

18. Julia C, Mejean C, Vicari F, Peneau S, Hercberg S. Public perception and characteristics related to acceptance of the sugar-sweetened beverage taxation launched in France in 2012. Public Health Nutr. 2015;**18**(14):2679-88 doi: 10.1017/s1368980014003231[published Online First: Epub Date]|.

19. Moretto N, Kendall E, Whitty J, et al. Yes, the government should tax soft drinks: findings from a citizens' jury in Australia. Int. J. Environ. Res. Public. Health 2014;**11**(3):2456-71 doi: 10.3390/ijerph110302456[published Online First: Epub Date]|.

20. Gollust SE, Barry CL, Niederdeppe J. Americans' opinions about policies to reduce consumption of sugar-sweetened beverages. Prev. Med. 2014;**63**:52-57 doi: http://dx.doi.org/10.1016/j.ypmed.2014.03.002[published Online First: Epub Date]|.

21. HM Government. Childhood Obesity: A Plan for Action, 2016.

22. Vanderlee L, Reid JL, White CM, et al. Evaluation of the online Beverage Frequency Questionnaire (BFQ). Nutr. J. 2018;**17**(1):73 doi: 10.1186/s12937-018-0380-8[published Online First: Epub Date]|.

23. Subar A, Kirkpatrick S, Mittl B, et al. The Automated Self-Administered 24-hour dietary recall (ASA24): a resource for researchers, clinicians, and educators from the National Cancer Institute. Journal of the Academy of Nutrition and Dietetics 2012;**112**(8):1134-7

24. Adams J, Bateman B, Becker F, et al. Effectiveness and acceptability of parental financial incentives and quasi-mandatory schemes for increasing uptake of vaccinations in preschool children: systematic review, qualitative study and discrete choice experiment. Health Technol. Assess. 2015;**19**(94):1-176 doi: 10.3310/hta19940[published Online First: Epub Date]|.

25. Mozaffarian D, Forouhi NG. Dietary guidelines and health—is nutrition science up to the task? BMJ 2018;**360**

26. Bhawra J, Reid J, White C, Vanderlee L, Raine K, Hammond D. Are young Canadians supportive of proposed nutrition policies and regulations? An overview of policy support and the impact of socio-demographic factors on public opinion. Can. J. Public Health. 2018;**doi: 10.17269/s41997-018-0066-1. [Epub ahead of print]**

27. Signal LN, Watts C, Murphy C, Eyles H, Ni Mhurchu C. Appetite for health-related food taxes: New Zealand stakeholder views. Health Promotion International 2017:dax019-dax19 doi: 10.1093/heapro/dax019[published Online First: Epub Date]|.

For peer review only - http://bmjopen.bmj.com/site/about/guidelines.xhtml

28. Nixon L, Mejia P, Cheyne A, Dorfman L. Big Soda's long shadow: news coverage of local proposals to tax sugar-sweetened beverages in Richmond, El Monte and Telluride. Critical Public Health 2015;**25**(3):333-47 doi: 10.1080/09581596.2014.987729[published Online First: Epub Date]|.

29. Promberger M, Dolan P, Marteau T. "Pay them if it works": discrete choice experiments on the acceptability of financial incentives to change health related behaviour. Social Science & Medicine` 2012;**75**(12):2509-14

For peer review only

Reporting checklist for cross sectional study.

Based on the STROBE cross sectional guidelines.

Instructions to authors

Complete this checklist by entering the page numbers from your manuscript where readers will find each of the items listed below.

Your article may not currently address all the items on the checklist. Please modify your text to include the missing information. If you are certain that an item does not apply, please write "n/a" and provide a short explanation.

Upload your completed checklist as an extra file when you submit to a journal.

In your methods section, say that you used the STROBE cross sectional reporting guidelines, and cite them as:

von Elm E, Altman DG, Egger M, Pocock SJ, Gotzsche PC, Vandenbroucke JP. The Strengthening the Reporting of Observational Studies in Epidemiology (STROBE) Statement: guidelines for reporting observational studies.

		Reporting Item	Page Number
Title	#1a	Indicate the study's design with a commonly used term in the title or the abstract	1
Abstract	#1b	Provide in the abstract an informative and balanced summary of what was done and what was found	2
Background / rationale	#2	Explain the scientific background and rationale for the investigation being reported	4-5
Objectives	#3	State specific objectives, including any prespecified hypotheses	5
Study design	#4	Present key elements of study design early in the paper	5-6
Setting	#5	Describe the setting, locations, and relevant dates, including periods of recruitment, exposure, follow-up, and data collection	5-6
Eligibility criteria	#6a	Give the eligibility criteria, and the sources and methods of selection of participants.	6

For peer review only

	#7	Clearly define all outcomes, exposures, predictors, potential confounders, and effect modifiers. Give diagnostic criteria, if applicable	6
Data sources / measurement	#8	For each variable of interest give sources of data and details of methods of assessment (measurement). Describe comparability of assessment methods if there is more than one group. Give information separately for for exposed and unexposed groups if applicable.	6
Bias	#9	Describe any efforts to address potential sources of bias	8
Study size	#10	Explain how the study size was arrived at	5-6
Quantitative variables	#11	Explain how quantitative variables were handled in the analyses. If applicable, describe which groupings were chosen, and why	8
Statistical methods	#12a	Describe all statistical methods, including those used to control for confounding	8
	#12b	Describe any methods used to examine subgroups and interactions	n/a
	#12c	Explain how missing data were addressed	6
	#12d	If applicable, describe analytical methods taking account of sampling strategy	8
	#12e	Describe any sensitivity analyses	n/a
Participants	#13a	Report numbers of individuals at each stage of study—eg numbers potentially eligible, examined for eligibility, confirmed eligible, included in the study, completing follow-up, and analysed. Give information separately for for exposed and unexposed groups if applicable.	8
	#13b	Give reasons for non-participation at each stage	8
	#13c	Consider use of a flow diagram	n/a
Descriptive data	#14a	Give characteristics of study participants (eg demographic, clinical, social) and information on exposures and potential confounders. Give information separately for exposed and unexposed groups if applicable.	10

For peer review only

	#14b	Indicate number of participants with missing data for each variable of interest	6
Outcome data	#15	Report numbers of outcome events or summary measures. Give information separately for exposed and unexposed groups if applicable.	11
Main results	#16a	Give unadjusted estimates and, if applicable, confounder-adjusted estimates and their precision (eg, 95% confidence interval). Make clear which confounders were adjusted for and why they were included	11
	#16b	Report category boundaries when continuous variables were categorized	7
	#16c	If relevant, consider translating estimates of relative risk into absolute risk for a meaningful time period	n/a
Other analyses	#17	Report other analyses done—e.g., analyses of subgroups and interactions, and sensitivity analyses	n/a
Key results	#18	Summarise key results with reference to study objectives	12
Limitations	#19	Discuss limitations of the study, taking into account sources of potential bias or imprecision. Discuss both direction and magnitude of any potential bias.	12-13
Interpretation	#20	Give a cautious overall interpretation considering objectives, limitations, multiplicity of analyses, results from similar studies, and other relevant evidence.	13-14
Generalisability	#21	Discuss the generalisability (external validity) of the study results	12
Funding	#22	Give the source of funding and the role of the funders for the present study and, if applicable, for the original study on which the present article is based	15

The STROBE checklist is distributed under the terms of the Creative Commons Attribution License CC-BY. This checklist was completed on 14. September 2018 using http://www.goodreports.org/, a tool made by the EQUATOR Network in collaboration with Penelope.ai

For peer review only

BMJ Open

SUPPORT FOR, AND PERCEIVED EFFECTIVENESS OF, THE UK SOFT DRINKS INDUSTRY LEVY AMONGST UK ADULTS: CROSS-SECTIONAL ANALYSIS OF THE INTERNATIONAL FOOD POLICY SURVEY

Journal:	*BMJ Open*
Manuscript ID	bmjopen-2018-026698.R1
Article Type:	Research
Date Submitted by the Author:	19-Nov-2018
Complete List of Authors:	Pell, David; University of Cambridge, Centre for Diet & Activity Research, MRC Epidemiology Unit<br>Penney, Tarra; University of Cambridge, Centre for Diet & Activity Research, MRC Epidemiology Unit<br>Hammond, D; University of Waterloo,<br>Vanderlee, Lana; University of Waterloo<br>White, Martin; University of Cambridge, Centre for Diet & Activity Research, MRC Epidemiology Unit<br>Adams, J; University of Cambridge, Centre for Diet & Activity Research
**Primary Subject Heading**:	Public health
Secondary Subject Heading:	Nutrition and metabolism
Keywords:	taxation, soda tax, PUBLIC HEALTH, attitudes, NUTRITION & DIETETICS

SCHOLARONE™
Manuscripts

SUPPORT FOR, AND PERCEIVED EFFECTIVENESS OF, THE UK SOFT DRINKS INDUSTRY LEVY AMONGST UK ADULTS: CROSS-SECTIONAL ANALYSIS OF THE INTERNATIONAL FOOD POLICY SURVEY

David Pell,[1] Tarra Penney,[1] David Hammond,[2] Lana Vanderlee,[2] Martin White,[1] Jean Adams[1]*

[1]Centre for Diet & Activity Research, MRC Epidemiology Unit, University of Cambridge, Cambridge, UK

[2]School of Public Health & Health Systems, University of Waterloo, Waterloo, Ontario, Canada

***Corresponding author:** Jean Adams, Centre for Diet & Activity Research, MRC Epidemiology Unit, University of Cambridge School of Clinical Medicine, Box 285 Institute of Metabolic Science, Cambridge Biomedical Campus, Cambridge, UK, CB2 0QQ. Email: jma79@medschl.cam.ac.uk Tel: +44 (0) 1223 769 142

Main text word count: 3039

For peer review only

For peer review only - http://bmjopen.bmj.com/site/about/guidelines.xhtml

ABSTRACT

Objectives To answer four questions: What are attitudes, knowledge and social norms around sugar-sweetened beverages (SSBs)? What are current levels of trust in messages on SSBs? What is current support for, and perceived effectiveness of, the UK Soft Drinks Industry Levy (SDIL)? What is the association between attitudes, knowledge, social norms, trust, SSB consumption and socio-demographic factors; and support for, and perceived effectiveness of, the SDIL?

Design Cross-sectional on-line survey.

Setting UK

Participants UK respondents to the 2017 International Food Policy Study aged 18-64 years who provided information on all variables of interest (n=3104).

Outcome measures Self-reported perceived effectiveness of, and support for, the SDIL.

Results Most participants supported the SDIL (70%), believed it will be effective (71%), had a positive attitude to SSBs (62%), had knowledge of the link between SSBs and obesity (90%), and trusted messages from health experts (61%), but not those from the food and beverage industry (73%). Nearly half (46%) had negative social norms about drinking SSBs. In adjusted models, older age, non-consumption of SSBs, social norms to not drinks SSBs, knowledge of the link between SSBs and obesity and trust in health expert messages were associated with greater support for the SDIL, whereas having dependent children and trusting messages from the food and beverage industry were associated with less support. In adjusted models, older age was associated with lower perceived effectiveness of the SDIL, whereas social norms to not drink SSBs, negative attitudes to SSBs, and trusting messages from health experts and the food and beverage industry were associated with greater perceived effectiveness.

Conclusions There was strong support for the SDIL and belief that it will be effective. Those with more 'public health' orientated norms and trust were generally more likely to support the SDIL or believe that it will be effective.

Keywords: taxation, soda tax, public health, attitudes, nutrition & dietetics

For peer review only - http://bmjopen.bmj.com/site/about/guidelines.xhtml

STRENGTHS AND LIMITATIONS OF THE STUDY

- We used a large, population representative sample.
- We were careful to present the SDIL as an intervention targeted at manufacturers rather than consumers, with revenues ear-marked for health-promotion activities.
- This is a cross-sectional analysis and we cannot be sure of the direction of causation between putative explanatory variables and outcomes.
- Whilst all have strong face validity, we have not explored other aspects of validity or reliability of any of the measures used; in many cases it would be hard to know what the 'gold standard' measure should be.
- A high proportion of participants who completed the survey were included in the analysis, but we do not know the response rate.

For peer review only

For peer review only - http://bmjopen.bmj.com/site/about/guidelines.xhtml

INTRODUCTION

In his March 2016 Budget Statement, the UK Chancellor of the Exchequer (minister of finance) announced a soft drinks industry levy (SDIL) to be implemented in April 2018.[1] The levy is imposed on industries importing or manufacturing sugar-sweetened beverages (SSBs) and includes two 'tiers'. Drinks with ≥8g of sugar per 100ml are charged £0.24 per litre and those with ≥5g but <8g per 100ml are charged £0.18 per litre. Alcoholic drinks, milk-based drinks and pure fruit juices are exempt irrespective of sugar content. The Chancellor stated that revenue raised would be spent on school sport and school breakfast clubs. An explicit aim of announcing the levy two years in advance of implementation, and defining two levy tiers, was to provide time for manufacturers to reformulate.[1] The nature and intent of the SDIL makes it unique amongst international SSB taxes.

The success or failure of policy interventions is often the result of actions and reactions by many stakeholders including government, civil society, industry, the health sector and consumers. In particular support for the SDIL may both be influenced by the SDIL and modify its effectiveness. More intrusive public health interventions, like food and tobacco taxes, generally receive lower levels of public support than less intrusive ones, like information giving.[2] Support for hypothetical SSB taxes has been reported to range from 36-60%.[3-17]

How a public health intervention is framed may also impact how acceptable it is to stakeholders. The SDIL is specifically framed as a levy on manufacturers, rather than consumers, and as a source of revenue for other health promoting purposes. The importance of framing interventions such that they redefine public health problems has been previously identified.[18] By specifically targeting manufacturers, the SDIL frames excessive SSB consumption, and the resultant health implications, as a problem of drinks manufacturers, rather than consumers. Support for hypothetical food taxes generally increases when it is proposed that the revenue raised would be used for health promoting purposes.[11 12 19 20] There is some wider evidence that public health messages in general framed in terms of gains, rather than losses, to recipients elicit more positive responses from the public.[21] Clearly stating that the SDIL is not targeted at consumers (and hence implying that consumers should not lose) and that revenues will be used for health promotion (and hence implying that consumers stand to gain) may, therefore, increase positive responses and hence support for it. Previous work has explored differences in support for SSB taxes according to participant socio-demographic characteristics, but findings are not consistent. For example, support has been varyingly reported as higher in younger people,[3 17 22] higher in older people,[19] and not associated with age.[5 10] Associations between support for SSB

For peer review only - http://bmjopen.bmj.com/site/about/guidelines.xhtml

taxes and both SSB consumption and markers of socio-economic position are similarly variable.[3 5 8 16 19 22] Fewer studies have explored psychological correlates of support for SSB taxes, such as attitudes, social norms, knowledge and trust. Those who felt that SSBs were a major (but not minor) contributor to childhood obesity in the USA were more likely to support an SSB tax.[5] Although trust in government was not associated with support in either the UK or USA,[10] more favourable assessments of soft drinks companies were associated with lower support in the USA.[22]

One reason for low support for SSB taxes commonly found in qualitative work is low perceived effectiveness of small changes in price.[6 7 11 12 20] Perceived effectiveness is less studied in quantitative studies, but has been found to range from 39-58%.[5 12 19] Perceived effectiveness was found to be an important correlate of support in one quantitative study,[10] and has also been reported to be higher in older people and those with more education; but lower in those consuming more SSBs.[19]

The great majority of work in this area has focused on hypothetical taxes. As support for more intrusive public health interventions often increases after implementation,[2] support for hypothetical SSB taxes may misrepresent support for taxes that have been announced or implemented. To date, we are aware of only one study that has explored public perceptions of a definite, rather than hypothetical, tax.[19] This study was conducted in France where an excise tax applies to all sweetened drinks, including those sweetened with artificial sweeteners. Given the difference between the French tax and SSB taxes, which are more specific to drinks sweetened with sugar, the French findings may not be generalisable.

In this study we explored both socio-demographic and psychological correlates of support for, and perceived effectiveness of, a definite, rather than hypothetical, SSB tax that has been framed in a unique way. Using data from UK adults collected 20 months after announcement and four months before implementation of the SDIL, our specific research questions were: 1. What are current attitudes, knowledge and social norms around SSBs? 2. What are current levels of trust in messages on SSBs from different institutions? 3. What is current support for, and perceived effectiveness of, the SDIL? 4. What is the association between attitudes, knowledge, social norms, trust, SSB consumption and socio-demographic factors; and support for, and perceived effectiveness of, the SDIL?

METHODS

The analyses were pre-specified in a protocol.

Sampling, recruitment and data collection

For peer review only - http://bmjopen.bmj.com/site/about/guidelines.xhtml

Data were from UK participants in Wave 1 of the International Food Policy Study, conducted in Australia, Canada, Mexico, the United Kingdom and the United States. Data were collected via self-completed web-based surveys in December 2017 with adults aged 18-64 years. Respondents were recruited through Nielsen Consumer Insights Global Panel and their partners' panels. Email invitations (with a unique link) were sent to a random sample of panelists (after targeting for age and country criteria); panelists known to be ineligible were not invited. The mean survey time across countries was 33 minutes.

Respondents provided consent prior to completing the survey. Respondents received remuneration in accordance with their panel's usual incentive structure (e.g., points-based or monetary rewards, or chances to win prizes). The study was reviewed by and received ethics clearance through a University of Waterloo Research Ethics Committee (ORE# 21460). A full description of the study methods can be found in the International Food Policy Study: Technical Report – Wave 1 (2017) at www.foodpolicystudy.com/methods.

Variables used in the analysis

The variables used in the analysis, the survey items they were derived from, response options and how response options were collapsed for analysis are described in Table 1.

Alongside single-item measures of attitudes, knowledge, and social norms related to sugary drinks; we included single items measures of trust in advice on sugary drinks from health experts and the food and beverage industry; and single item measures of support for, and perceived effectiveness of, the SDIL. As previous research has indicated that the acceptability of food taxes varies with the stated intentions of these,[11 12 19 20] we included a preamble to the questions about support for, and perceived effectiveness of, the SDIL outlining the intention of the levy and the stated use of revenue generated.

Sociodemographic variables considered were age in years, sex at birth, whether or not participants had children and socio-economic position. Parental status was a potentially important variable because the SDIL is included as a flagship component of England's Childhood Obesity Plan and has particularly been framed in terms of potential benefits to children.[1 23] Socio-economic position was measured using participants' highest educational qualification and perceived income sufficiency.

The BFQ is a 7-day food record that assesses consumption for 17 beverage categories, including caloric and non-caloric beverages.[24] For each beverage category, respondents report the number of drinks and the usual portion size, using category-specific images of beverage containers, adapted from the

For peer review only - http://bmjopen.bmj.com/site/about/guidelines.xhtml

ASA24 dietary recall.[25] Participants who reported any consumption of regular fizzy drinks (including alcoholic drinks that contained regular fizzy drinks as a mixer), sweetened fruit drinks, sports drinks, or energy drinks over the previous seven days were considered SSB consumers in the analysis.

Inclusion criteria

UK resident participants in wave 1 of the International Food Policy Survey, aged 18-64 years, who correctly responding to a data integrity question in which participants were asked to identify the current month, and provided usable information on all other variables of interest were included in the analysis. Data from countries other than the UK were not included as comparable questions on support for, and perceived effectiveness of, the SDIL were not asked of participants from these countries.

For peer review only

For peer review only - http://bmjopen.bmj.com/site/about/guidelines.xhtml

Table 1. Description of items and response options used in the analysis

Concept	Item wording (where applicable)	Response options: All	Response options: Used in analysis
Age	How old are you?	In years	In years
Sex	What sex were you assigned at birth, meaning on your original birth certificate?	Female	Female
		Male	Male
Education	What is the highest level of education you have completed?	Qualifications not listed below, free-text equivalents, Don't Know, Refuse to answer	≤School leaving
		NVQ Level 4-5, HNC, HND, RSA Higher Diploma, BTEC Higher Level, Degree, Higher Degree, free-text equivalents	>School leaving
Income sufficiency	How easy is it to make ends meet?	Neither easy nor difficult, Difficult, Very difficult, Don't know, Refuse to answer	Not easy
		Very easy, Easy	Easy
Children	Do you have any children (including step-children or adopted children) under the age of 18?	No, Don't know, Refuse to answer	No
		Yes	Yes
SSB consumption	[Calculated from Beverage Frequency Questionnaire: reported consumption over last 7 days]	Any consumption of non-diet Fizzy drinks, Sweetened fruit juice drinks, Regular sports drinks, Regular energy drinks, or Spirits with mixers that have calories	Consumers
		No consumption of above	Non-consumers
Social norms	People important to me try not to drink sugary drinks	Neither agree nor disagree, Disagree, Strongly disagree, Don't know, Refuse to answer	Not agree
		Strongly agree, Agree	Agree
Attitudes	Sugary drinks taste good	Strongly agree, Agree	Agree
		Neither agree nor disagree, Disagree, Strongly disagree, Don't know, Refuse to answer	Not agree
Knowledge	Frequently drinking sugary drinks increases the risk of obesity	False, Don't know, Refuse to answer	Not true
		True	True
Expert trust	I trust messages from health experts on sugary drinks	Neither agree nor disagree, Disagree, Strongly disagree, Don't know, Refuse to answer	Not agree
		Strongly agree, Agree	Agree
Industry trust	I trust messages from the food and beverage industry on sugary drinks?	Neither agree nor disagree, Disagree, Strongly disagree, Don't know, Refuse to answer	Not agree
		Strongly agree, Agree	Agree
Support	In 2018 a new sugary drink tax will be introduced in the UK. This aims to encourage manufacturers to reduce the sugar in drinks. The money will be spent on breakfast clubs, and sports in primary schools. Do you support or oppose this policy?	Strongly support, Support	Support
		Oppose, Strongly oppose, Don't know, Refuse to answer	Oppose
Effectiveness	Preamble as above. How effective do you think these kinds of policies are?	Somewhat effective, Mostly effective, Very effective	Effective
		Not at all effective, Don't know, Refuse to answer	Not effective

For peer review only

For peer review only - http://bmjopen.bmj.com/site/about/guidelines.xhtml

Analysis

Data were weighted with post-stratification sample weights constructed using population estimates from the UK census based on age group, sex and region. These sample weights were used throughout the analysis to reduce the effects of non-response and selection bias and return the sample to population representativeness.

Descriptive statistics were used to quantify all variables of interest. Logistic regression models were fitted to explore associations between other variables and support for, and perceived effectiveness of, the SDIL. We used separate models to explore support for the SDIL and perceived effectiveness of the SDIL where support for, or perceived effectiveness of, the SDIL were the outcome variables and all other variables were included as explanatory variables. Unless otherwise noted, adjusted odds ratios (and 95% confidence intervals) of support for, or perceived effectiveness of, the SDIL are presented adjusted for all other variables included.

Data were analysed using R version 3.3.1.

Patient and public involvement

Patients and the public were not involved in design, conduct, analysis or interpretation of the study.

RESULTS

Of 4276 who took part in the in the UK arm of the International Food Policy Survey in December 2017, 4047 (95%) correctly responded to the data integrity question. Of these, 3104 (77%) provided complete data on all variables of interest and were included the analysis.

Characteristics of the analytical sample (after applying survey weights) are described in Table 2. Participants had a mean age of 38 (standard deviation 13) years, with a good balance across sex at birth (48% female). The highest level of education that most participants had achieved was the equivalent of school-leaving or lower and around two thirds (61%) did not find it easy to make ends meet. Just over one third (37%) of participants had children under the age of 18 years, and just less than half (47%) reported consuming SSBs in the last seven days.

Around half of participants (54%) agreed that people important to them try not to drink SSBs (social norms), around two thirds (62%) that SSBs taste good (attitudes), and 90% believed that frequently consuming SSBs increases the risk of obesity (knowledge). Whilst more than half (61%) of respondents

For peer review only - http://bmjopen.bmj.com/site/about/guidelines.xhtml

For peer review only

trusted messages from health experts on SSBs, only one quarter (27%) trusted messages from the food and beverage industry.

Table 3 shows the results of logistic regression analyses of associations between socio-demographics, social norms, attitudes, knowledge and trust, and perceived support for, and effectiveness of, the SDIL – adjusted for all other variables in the models.

In adjusted models, older participants were more likely to support the SDIL, but were less likely to consider it effective. Those with dependent children and those who trusted messages from the food and beverage industry on sugary drinks were less likely to support the SDIL. Non-consumers of SSBs, those with social norms to not drinks SSBs, those with knowledge of the association between SSBs and obesity, and those who trust messages from health experts on sugary drinks were more likely to support the SDIL than other. Those with high social norms around not drinking SSBs, less positive attitudes to sugary drinks, and those who trusted messages on sugary drinks from health experts and from the food and beverage industry were more likely to consider the SDIL would be effective. There were no differences in support for or perceived effectiveness of the SDIL by sex, education or perceived income sufficiency.

For peer review only

For peer review only - http://bmjopen.bmj.com/site/about/guidelines.xhtml

Table 2. Weighted characteristics of UK participants in the International Food Policy Survey, Dec 2017 (N = 3104)

Concept	Question wording (where applicable)	Response category	n	%
Sex	What sex were you assigned at birth, meaning on your original birth certificate?	Female	1497	48
Education	What is the highest level of education you have completed?	A-Levels or lower	1896	61
Income sufficiency	How easy is it to make ends meet?	Not easy	1905	61
Children	Do you have any children (including step-children or adopted children) under the age of 18?	No	1963	63
SSB consumption	Consumed regular fizzy drinks, sweetened fruit drinks, sports drinks, energy drinks in last 7 days	Consumers	1473	47
Social norms	People important to me try not to drink sugary drinks	Not agree	1416	46
Attitudes	Sugary drinks taste good	Agree	1938	62
Knowledge	Frequently drinking sugary drinks increases the risk of obesity	Not true	322	10
Expert trust	I trust messages from health experts on sugary drinks	Not agree	1213	39
Industry trust	I trust messages from the food and beverage industry on sugary drinks	Not agree	2267	73
Support	In 2018 a new sugary drink tax will be introduced in the UK. This aims to encourage manufacturers to reduce the sugar in drinks. The money will be spent on breakfast clubs, and sports in primary schools. Do you support or oppose this policy?	Support	2167	70
Effectiveness	Preamble as above. How effective do you think these kinds of policies are?	Effective	2214	71

Table 3. Adjusted* odds ratios (95% confidence intervals) of characteristics associated with support for, and perceived effectiveness of, the SDIL

Concept	Question wording (where applicable)	Response category	Adjusted OR (95% CI) of SDIL support	Adjusted OR (95% CI) of SDIL effectiveness
Age	How old are you?	Years	**1.01 (1.00 to 1.02)**	**0.99 (0.98 to 0.99)**
Sex	What sex were you assigned at birth, meaning on your original birth certificate?	Female	Reference	Reference
		Male	(0.72 to 1.05)	1.03 (0.85 to 1.25)
Education	What is the highest level of education you have completed?	A-Levels or lower	Reference	Reference
		> A-Levels	1.03 (0.85 to 1.26)	0.90 (0.73 to 1.10)
Income sufficiency	How easy is it to make ends meet?	Not easy	Reference	Reference
		Easy	1.01 (0.83 to 1.24)	1.02 (0.83 to 1.25)

For peer review only - http://bmjopen.bmj.com/site/about/guidelines.xhtml

Dependent children	Do you have any children (including step-children or adopted children) under the age of 18?	No	Reference	Reference
		Yes	**0.81 (0.67 to 0.99)**	1.16 (0.94 to 1.43)
SSB consumption	Consumed regular fizzy drinks, sweetened fruit drinks, sports drinks, energy drinks in last 7 days	Consumers	Reference	Reference
		Non-consumers	**1.57 (1.28 to 1.91)**	1.21 (0.99 to 1.48)
Social norms	People important to me try not to drink sugary drinks	Not agree	Reference	Reference
		Agree	**1.39 (1.15 to 1.70)**	**1.25 (1.03 to 1.53)**
Attitudes	Sugary drinks taste good	Agree	Reference	Reference
		Not agree	1.10 (0.89 to 1.36)	**1.31 (1.07 to 1.61)**
Knowledge	Frequently drinking sugary drinks increases the risk of obesity	Not true	Reference	Reference
		True	**2.34 (1.74 to 3.16)**	1.06 (0.77 to 1.45)
Expert trust	I trust messages from health experts on sugary drinks	Not agree	Reference	Reference
		Agree	**2.01 (1.63 to 2.49)**	**1.86 (1.51 to 2.28)**
Industry trust	I trust messages from the food and beverage industry on sugary drinks	Not agree	Reference	Reference
		Agree	**0.55 (0.44 to 0.69)**	**1.37 (1.08 to 1.75)**

*All results are adjusted for all other variables listed; SDIL = soft drinks industry levy; **BOLD** indicates statistically significant at the $p<0.05$ level

For peer review only - http://bmjopen.bmj.com/site/about/guidelines.xhtml

DISCUSSION

Summary of findings

To our knowledge, this is the first study of a range of socio-demographic, consumption and psychological correlates of both support for, and perceived effectiveness of, an SSB tax. Unlike previous studies, our research was conducted in the context of a definite, rather than hypothetical, SSB tax. We found that the majority of UK adults aged 18-64 years were supportive of the SDIL and believe it will be effective, have a positive attitude to SSBs, have good knowledge about the links between SSBs and obesity, and trust messages from health experts, but not the food and beverage industry, about sugary drinks. Around half reported social norms about not drinking SSBs.

Social norms towards not consuming SSBs and trusting health expert messages on SSBs were both associated with greater support for and perceived effectiveness of the SDIL. In addition, having dependent children and trusting messages from the food and beverage industry on sugary drinks were associated with less support for the SDIL, whilst older age, not consuming SSBs and knowledge of the link between sugary drinks and obesity were associated with greater support. Older age was associated with lower perceived effectiveness of the SDIL, and more negative attitudes towards sugary drinks were associated with greater perceived effectiveness. There were no associations between gender, education or income sufficiency and either support for, of perceived effectiveness of, the SDIL.

Strengths and weaknesses of methods

Key strengths of the analysis are the use of a large, population representative, sample; inclusion of a range of socio-demographic, consumption and psychological variables; and the context of a definite, rather than hypothetical, SSB tax announced 20 months before data collection (although not implemented until four months after). Given previous findings that support is greater when revenues are used for health-promoting activities,[11 12 19 20] we were careful to present the SDIL as an intervention targeting manufacturers rather than consumers, with revenues ear-marked for health-promotion activities. Social desirability bias may be less likely to occur in more anonymous settings such as on-line surveys.[26]

Participants were not recruited using probability-based sampling meaning the findings do not provide nationally representative estimate, although this was reduced by applying sampling weights. The results are, therefore, likely to be generalizable to the UK, but may not be more widely generalizable. This is a cross-sectional analysis and we cannot be sure of the direction of causation between putative

For peer review only - http://bmjopen.bmj.com/site/about/guidelines.xhtml

explanatory variables and outcomes. Nor have we explored more complicated causal networks linking the variables included. All variables were self-reported. Whilst all have strong face validity, we have not explored other aspects of validity or reliability of any of the measures used. However, all were derived from existing instruments in some cases it would be hard to know what a 'gold standard' measure should be. Although a high proportion of participants who completed the survey were included in the analysis, we do not know what proportion of those invited to participate were included.

Comparison to previous results and interpretation of findings

Most people in our survey (90%) knew that there was an association between SSB consumption and obesity. This reflects previous findings where 89-91% agreed that SSB consumption increased the risk of obesity.[3 5] Despite this, there were also high positive attitudes towards SSBs with almost two-third of respondents agreeing that sugary drinks taste good, and less than half had social norms about not drinking SSBs. In the UK, SSBs appear to remain a pleasurable and positive part of life, despite their known health harms.

Similar to previous research which found that only 30% of Americans gave favourable ratings to soda companies,[22] we found low levels of trust in messages about SSBs from the food and beverage industry. Levels of trust in similar messages from health experts were higher, but still less than two thirds. Low levels of trust in experts may reflect a general public mistrust of nutritional epidemiology.[27]

Despite less than perfect trust in messages about SSBs from health experts, there was a high level of support for the SDIL (70%) and even higher belief that it would be effective (71%). This is higher than previous research which, as far as we are aware, reports maximum support of 60%.[8 28] Even in the context of an existing tax on sweetened drinks in France, only 49% supported the tax.[19] The high level of support we found may reflect the combined effect of previous findings that support for more intrusive public health interventions such as taxes on food and tobacco often increases after implementation,[2] and that support for SSB taxes is often greater when revenues are used for health-promoting activities.[11 12 19 20 28 29] Although the SDIL had not been implemented at the time of data collection, impending implementation had been known of for 20 months. Further, we were careful to inform participants that SDIL revenues would be spent on school breakfast clubs and sports activities. In addition, the SDIL is unique in being targeted at manufacturers rather than consumers, and intended to promote reformulation rather than necessarily reduce consumption.[1] Previous qualitative work has found that those who do not support generic SSB taxes often cite excessive personal taxation and

For peer review only - http://bmjopen.bmj.com/site/about/guidelines.xhtml

government intrusion into individual's lives as reasons for this.[11 30] This is much less applicable to the SDIL than to consumer-facing SSB taxes.

Low acceptability of SSB taxes has previously been ascribed to a perception that they are unlikely to achieve significant behaviour change or public health benefit.[11 30] Previous research has reported perceived effectiveness (to improve population health or decrease SSB consumption) in the range of 39-58%.[5 12 19] In contrast, we found much higher levels of perceived effectiveness (71%). This may again reflect the unique nature of the SDIL with an explicit intention to change manufacturer, rather than consumer, behaviour – and our focus on effects on industry, rather than consumer, behaviour.

Higher support for, and perceived effectiveness of, the SDIL here compared to previous work may also reflect cultural differences between the UK and other countries where previous data has been collected. Unlike previously, we used population weighting which increases confidence that results are population representative. Finally, it is possible that the unique design and framing of the SDIL makes it more acceptable and increases perceived effectiveness compared to previous taxes proposed to research participants.

The pattern of associations between attitudes, social norms, trust and support for, and perceived effectiveness of, the SDIL are, for the most part, intuitive. It might be expected that non-consumers, who are less likely to be negatively financially effected by the tax, would be more supportive. In other contexts, those who stand to gain most from financial incentive interventions are most supportive.[31] Social norms to not drink sugary drinks, negative attitudes towards sugary drinks, greater knowledge about the health harms of sugary drinks, greater trust in health experts and less trust in the food and beverage industry all reflect more 'public health' orientated patterns that would be expected to be associated with greater support for, or perceived effectiveness, of the SDIL. It is somewhat surprising that those with children under the age of 18 years were less supportive of the SDIL than those without. The SDIL was particularly framed in terms of potential benefits to children.[1 23] If one's own consumption is likely to influence support for the SDIL, then parents' support for the SDIL may also be influenced by their children's consumption. If children are greater consumers of sugary drinks,[32] then this may explain why parents with children under the age of 18 years were less supportive. As described above, previous research on the association between psychological variables and support for, and perceived effectiveness of, SSB taxes is sparse.

We did not find that gender or markers of socio-economic position were associated with support for, or perceived effectiveness of, the SDIL in mutually adjusted models. This reflects some, but not all,

For peer review only - http://bmjopen.bmj.com/site/about/guidelines.xhtml

previous findings.[5 8 10 19 22] Unlike most previous work we included a wide range of socio-demographic, consumption and psychological variables in mutually adjusted models and it may be that gender or socio-economic differences operate entirely through the other variables included in our models.

Implications of findings

Many structural public health policies require government action, which may be limited by perceptions concerning public acceptability of such policies – often uninformed by evidence. Greater understanding of public acceptability of a range of structural public health policies, and how this changes over time and the course of implementation, may help to develop strategies to address public concerns and build public support.

CONCLUSIONS

UK adults tend to have positive attitudes to SSBs and do not necessarily have strong social norms about not drinking SSBs, but they generally recognise the link between SSB consumption and obesity. Trust in messages about SSBs from the food and drinks industry is low, but trust in these messages from health experts is not universally high. There was strong support for the SDIL and belief that it will be effective. Those with more 'public health' orientated norms and trust were generally more likely to support the SDIL or believe that it will be effective, although those with dependent children were less likely to support the SDIL.

FUNDING

Funding for the International Food Policy Study was provided by the Canadian Institutes of Health Research (CIHR; operating grant). Additional support was provided by a CIHR – Public Health agency of Canada (PHAC) Applied Public Health Research Chair. The study has no affiliations with the food industry. The analyses reported in this paper were supported by The Health Foundation. JA & MW are supported by the Centre for Diet and Activity Research (CEDAR), a UKCRC Public Health Research Centre of Excellence. Funding from the British Heart Foundation, Cancer Research UK, Economic and Social Research Council, Medical Research Council, the National Institute for Health Research, and the Wellcome Trust, under the auspices of the UK Clinical Research Collaboration, is gratefully acknowledged (grant number MR/K023187/1). Views expressed in this paper are those of the authors and not necessarily those of the above named funders.

For peer review only - http://bmjopen.bmj.com/site/about/guidelines.xhtml

COMPETING INTERESTS

None declared

AUTHOR STATEMENT

JA, TP & MW conceived the idea for this paper. DP analysed the data. JA drafted the manuscript. JA, TP, MW, DH and LV read and provided critical comments on the manuscript and approved the final version. DH conceived the idea for the IFPS and secured funding. DH and LV developed the first draft of survey. TP led the further development of the UK survey instrument, with input from JA, MW, DH and LV.

DATA SHARING

Data is available directly from the International Food Policy Study team on reasonable request (see www.foodpolicystudy.com).

For peer review only

For peer review only - http://bmjopen.bmj.com/site/about/guidelines.xhtml

REFERENCES

1. Gauke D. Budget 2016. London: HM Treasury, 2016.

2. Diepeveen S, Ling T, Suhrcke M, Roland M, Marteau T. Public acceptability of government intervention to change health-related behaviours: a systematic review and narrative synthesis. BMC Public Health 2013;**13**:756

3. Rivard C, Smith D, McCann SE, Hyland A. Taxing sugar-sweetened beverages: a survey of knowledge, attitudes and behaviours. Public Health Nutr. 2012;**15**(8):1355-61 doi: 10.1017/s1368980011002898[published Online First: Epub Date]|.

4. Barry CL, Niederdeppe J, Gollust SE. Taxes on sugar-sweetened beverages: results from a 2011 national public opinion survey. Am. J. Prev. Med. 2013;**44**(2):158-63 doi: 10.1016/j.amepre.2012.09.065[published Online First: Epub Date]|.

5. Donaldson EA, Cohen JE, Rutkow L, Villanti AC, Kanarek NF, Barry CL. Public support for a sugar-sweetened beverage tax and pro-tax messages in a Mid-Atlantic US state. Public Health Nutr. 2015;**18**(12):2263-73

6. Jou J, Niederdeppe J, Barry CL, Gollust SE. Strategic messaging to promote taxation of sugar-sweetened beverages: lessons from recent political campaigns. Am. J. Public Health 2014;**104**(5):847-53 doi: 10.2105/ajph.2013.301679[published Online First: Epub Date]|.

7. Niederdeppe J, Gollust SE, Jarlenski MP, Nathanson AM, Barry CL. News coverage of sugar-sweetened beverage taxes: pro- and antitax arguments in public discourse. Am. J. Public Health 2013;**103**(6):e92-8 doi: 10.2105/ajph.2012.301023[published Online First: Epub Date]|.

8. Simon PA, Chiang C, Lightstone AS, Shih M. Public Opinion on Nutrition-Related Policies to Combat Child Obesity, Los Angeles County, 2011. Prev. Chronic. Dis. 2014;**11**:E96 doi: 10.5888/pcd11.140005[published Online First: Epub Date]|.

9. Somerville C, Marteau TM, Kinmonth AL, Cohn S. Public attitudes towards pricing policies to change health-related behaviours: a UK focus group study. The European Journal of Public Health 2015 doi: 10.1093/eurpub/ckv077[published Online First: Epub Date]|.

10. Petrescu D, Hollands G, Couturier D, Ng Y, Marteau T. Public acceptability in the UK and USA of nudging to reduce obesity: The example of reducing sugar-sweetened beverages consumption. PLoS ONE 2016;**11**(6):1-18

11. Thomas-Meyer M, Mytton O, Adams J. Public responses to proposals for a tax on sugar-sweetened beverages: A thematic analysis of online reader comments posted on major UK news websites. PLoS ONE 2017;**12**(11):e0186750 doi: 10.1371/journal.pone.0186750[published Online First: Epub Date]|.

12. Timpson H, Lavin R, Hughes L. Exploring the Acceptability of a Tax on Sugar-Sweetened Beverages: Insight Work. Liverpool: Centre for Public Health, Liverpool John Moores University, 2013.

13. Beeken RJ, Wardle J. Public beliefs about the causes of obesity and attitudes towards policy initiatives in Great Britain. Public Health Nutr. 2013;**16**(12):2132-7 doi: 10.1017/s1368980013001821[published Online First: Epub Date]|.

For peer review only - http://bmjopen.bmj.com/site/about/guidelines.xhtml

14. Cancer Research UK, UK Health Forum. Short and sweet: why the government should introduce a sugary drinks tax in the UK. London, 2016.

15. ComRes. Daily Mail sugar tax poll. Secondary Daily Mail sugar tax poll 2015. http://www.comres.co.uk/polls/daily-mail-sugar-tax-poll/.

16. Ipsos Mori Social Research Institute. Great Britain: The way we live now. Understanding Society 2013;**December 2013**

17. Curry LE, Rogers T, Williams P, Homsi G, Willett J, Schmitt CL. Public Attitudes and Support for a Sugar-Sweetened Beverage Tax in America's Heartland. Health Promot. Pract. 2017:1524839917709759 doi: 10.1177/1524839917709759[published Online First: Epub Date]|.

18. Randolph W, Viswanath K. Lessons Learned from Public Health Mass Media Campaigns: Marketing Health in a Crowded Media World. Annu. Rev. Public Health 2004;**25**(1):419-37 doi: 10.1146/annurev.publhealth.25.101802.123046[published Online First: Epub Date]|.

19. Julia C, Mejean C, Vicari F, Peneau S, Hercberg S. Public perception and characteristics related to acceptance of the sugar-sweetened beverage taxation launched in France in 2012. Public Health Nutr. 2015;**18**(14):2679-88 doi: 10.1017/s1368980014003231[published Online First: Epub Date]|.

20. Moretto N, Kendall E, Whitty J, et al. Yes, the government should tax soft drinks: findings from a citizens' jury in Australia. Int. J. Environ. Res. Public. Health 2014;**11**(3):2456-71 doi: 10.3390/ijerph110302456[published Online First: Epub Date]|.

21. Shen L, Dillard JP. The Influence of Behavioral Inhibition/Approach Systems and Message Framing on the Processing of Persuasive Health Messages. Communication Research 2007;**34**(4):433-67 doi: 10.1177/0093650207302787[published Online First: Epub Date]|.

22. Gollust SE, Barry CL, Niederdeppe J. Americans' opinions about policies to reduce consumption of sugar-sweetened beverages. Prev. Med. 2014;**63**:52-57 doi: http://dx.doi.org/10.1016/j.ypmed.2014.03.002[published Online First: Epub Date]|.

23. HM Government. Childhood Obesity: A Plan for Action, 2016.

24. Vanderlee L, Reid JL, White CM, et al. Evaluation of the online Beverage Frequency Questionnaire (BFQ). Nutr. J. 2018;**17**(1):73 doi: 10.1186/s12937-018-0380-8[published Online First: Epub Date]|.

25. Subar A, Kirkpatrick S, Mittl B, et al. The Automated Self-Administered 24-hour dietary recall (ASA24): a resource for researchers, clinicians, and educators from the National Cancer Institute. Journal of the Academy of Nutrition and Dietetics 2012;**112**(8):1134-7

26. Adams J, Bateman B, Becker F, et al. Effectiveness and acceptability of parental financial incentives and quasi-mandatory schemes for increasing uptake of vaccinations in preschool children: systematic review, qualitative study and discrete choice experiment. Health Technol. Assess. 2015;**19**(94):1-176 doi: 10.3310/hta19940[published Online First: Epub Date]|.

27. Mozaffarian D, Forouhi NG. Dietary guidelines and health—is nutrition science up to the task? BMJ 2018;**360**

For peer review only

For peer review only - http://bmjopen.bmj.com/site/about/guidelines.xhtml

28. Bhawra J, Reid J, White C, Vanderlee L, Raine K, Hammond D. Are young Canadians supportive of proposed nutrition policies and regulations? An overview of policy support and the impact of socio-demographic factors on public opinion. Can. J. Public Health. 2018;**doi: 10.17269/s41997-018-0066-1. [Epub ahead of print]**

29. Signal LN, Watts C, Murphy C, Eyles H, Ni Mhurchu C. Appetite for health-related food taxes: New Zealand stakeholder views. Health Promotion International 2017:dax019-dax19 doi: 10.1093/heapro/dax019[published Online First: Epub Date]|.

30. Nixon L, Mejia P, Cheyne A, Dorfman L. Big Soda's long shadow: news coverage of local proposals to tax sugar-sweetened beverages in Richmond, El Monte and Telluride. Critical Public Health 2015;**25**(3):333-47 doi: 10.1080/09581596.2014.987729[published Online First: Epub Date]|.

31. Promberger M, Dolan P, Marteau T. "Pay them if it works": discrete choice experiments on the acceptability of financial incentives to change health related behaviour. Social Science & Medicine` 2012;**75**(12):2509-14

32. Ng SW, Ni Mhurchu C, Jebb SA, Popkin BM. Patterns and trends of beverage consumption among children and adults in Great Britain, 1986–2009. Br. J. Nutr. 2012;**108**(03):536-51 doi: doi:10.1017/S0007114511006465[published Online First: Epub Date]|.

For peer review only

Reporting checklist for cross sectional study.

Based on the STROBE cross sectional guidelines.

Instructions to authors

Complete this checklist by entering the page numbers from your manuscript where readers will find each of the items listed below.

Your article may not currently address all the items on the checklist. Please modify your text to include the missing information. If you are certain that an item does not apply, please write "n/a" and provide a short explanation.

Upload your completed checklist as an extra file when you submit to a journal.

In your methods section, say that you used the STROBE cross sectional reporting guidelines, and cite them as:

von Elm E, Altman DG, Egger M, Pocock SJ, Gotzsche PC, Vandenbroucke JP. The Strengthening the Reporting of Observational Studies in Epidemiology (STROBE) Statement: guidelines for reporting observational studies.

		Reporting Item	Page Number
Title	#1a	Indicate the study’s design with a commonly used term in the title or the abstract	1
Abstract	#1b	Provide in the abstract an informative and balanced summary of what was done and what was found	2
Background / rationale	#2	Explain the scientific background and rationale for the investigation being reported	4-5
Objectives	#3	State specific objectives, including any prespecified hypotheses	5
Study design	#4	Present key elements of study design early in the paper	5-6
Setting	#5	Describe the setting, locations, and relevant dates, including periods of recruitment, exposure, follow-up, and data collection	5-6
Eligibility criteria	#6a	Give the eligibility criteria, and the sources and methods of selection of participants.	6

For peer review only

	#7	Clearly define all outcomes, exposures, predictors, potential confounders, and effect modifiers. Give diagnostic criteria, if applicable	6
Data sources / measurement	#8	For each variable of interest give sources of data and details of methods of assessment (measurement). Describe comparability of assessment methods if there is more than one group. Give information separately for for exposed and unexposed groups if applicable.	6
Bias	#9	Describe any efforts to address potential sources of bias	8
Study size	#10	Explain how the study size was arrived at	5-6
Quantitative variables	#11	Explain how quantitative variables were handled in the analyses. If applicable, describe which groupings were chosen, and why	8
Statistical methods	#12a	Describe all statistical methods, including those used to control for confounding	8
	#12b	Describe any methods used to examine subgroups and interactions	n/a
	#12c	Explain how missing data were addressed	6
	#12d	If applicable, describe analytical methods taking account of sampling strategy	8
	#12e	Describe any sensitivity analyses	n/a
Participants	#13a	Report numbers of individuals at each stage of study—eg numbers potentially eligible, examined for eligibility, confirmed eligible, included in the study, completing follow-up, and analysed. Give information separately for for exposed and unexposed groups if applicable.	8
	#13b	Give reasons for non-participation at each stage	8
	#13c	Consider use of a flow diagram	n/a
Descriptive data	#14a	Give characteristics of study participants (eg demographic, clinical, social) and information on exposures and potential confounders. Give information separately for exposed and unexposed groups if applicable.	10

For peer review only

	#14b	Indicate number of participants with missing data for each variable of interest	6
Outcome data	#15	Report numbers of outcome events or summary measures. Give information separately for exposed and unexposed groups if applicable.	11
Main results	#16a	Give unadjusted estimates and, if applicable, confounder-adjusted estimates and their precision (eg, 95% confidence interval). Make clear which confounders were adjusted for and why they were included	11
	#16b	Report category boundaries when continuous variables were categorized	7
	#16c	If relevant, consider translating estimates of relative risk into absolute risk for a meaningful time period	n/a
Other analyses	#17	Report other analyses done—e.g., analyses of subgroups and interactions, and sensitivity analyses	n/a
Key results	#18	Summarise key results with reference to study objectives	12
Limitations	#19	Discuss limitations of the study, taking into account sources of potential bias or imprecision. Discuss both direction and magnitude of any potential bias.	12-13
Interpretation	#20	Give a cautious overall interpretation considering objectives, limitations, multiplicity of analyses, results from similar studies, and other relevant evidence.	13-14
Generalisability	#21	Discuss the generalisability (external validity) of the study results	12
Funding	#22	Give the source of funding and the role of the funders for the present study and, if applicable, for the original study on which the present article is based	15

The STROBE checklist is distributed under the terms of the Creative Commons Attribution License CC-BY. This checklist was completed on 14. September 2018 using http://www.goodreports.org/, a tool made by the EQUATOR Network in collaboration with Penelope.ai

For peer review only
